# Chlorogenic Acid Restores Ovarian Functions in Mice with Letrozole-Induced Polycystic Ovarian Syndrome Via Modulation of Adiponectin Receptor

**DOI:** 10.3390/biomedicines11030900

**Published:** 2023-03-14

**Authors:** Mohd Zahoor ul Haq Shah, Vinoy Kumar Shrivastava, Shazia Sofi, Yahya F. Jamous, Mohd Faiyaz Khan, Faisal K. Alkholifi, Wasim Ahmad, Manzoor Ahmad Mir

**Affiliations:** 1 Endocrinology Unit, Department of Bioscience, Barkatullah University, Bhopal 462026, India; scholarendocrinology@gmail.com (M.Z.u.H.S.);; 2 Department of Bioresources, School of Biological Sciences, University of Kashmir Srinagar, Srinagar 190006, India; 3 Vaccine and Bioprocessing Centre, King Abdulaziz City for Science and Technology (KACST), Riyadh 11442, Saudi Arabia; 4 Department of Clinical Pharmacy, College of Pharmacy, Prince Sattam Bin Abdulaziz University, Al-kharj 16278, Saudi Arabia; 5 Department of Pharmacology, College of Pharmacy, Prince Sattam Bin Abdulaziz University, Al-kharj 16278, Saudi Arabia; 6 Department of Pharmacy, Mohammad Al-Mana College for Medical Sciences, Damman 34222, Saudi Arabia

**Keywords:** chlorogenic acid, PCOS, adiponectin, letrozole, oxidative stress, inflammation, insulin resistance

## Abstract

Around the world, polycystic ovary syndrome (PCOS) is a complex endocrine-metabolic condition that typically affects 6–20% of females. Our study’s major goal was to examine how chlorogenic acid (CGA) affected mice with endocrine and metabolic problems brought on by letrozole-induced PCOS. Group I served as the control for 81 days; Group II was given Letrozole (LETZ) orally at a dose of 6 mg/kg bw for 21 days to induce PCOS; Group III was given LETZ (6 mg/kg) for 21 days, followed by treatment with CGA (50 mg/kg bw daily) for 60 days. The study indicated that LETZ-treated mice displayed symptoms of PCOS, such as dyslipidemia, hyperinsulinemia, elevated testosterone, increases in inflammatory markers and malonaldehyde, and a decline in antioxidants (Ar, lhr, fshr, and esr2) in the ovaries. These alterations were affected when the mice were given CGA and were associated with reduced levels of adiponectin. Adiponectin showed interactions with hub genes, namely MLX interacting protein like (MLXIPL), peroxisome proliferator-activated receptor gamma Coactivator 1- alpha (PPARGC1), peroxisome proliferator-activated receptor gamma (Pparg), and adiponectin receptor 1 (Adipor1). Lastly, the gene ontology of adiponectin revealed that adiponectin was highly involved in biological processes. The findings from our research suggest that adiponectin has direct impacts on metabolic and endocrine facets of PCOS.

## 1. Introduction

Polycystic ovary syndrome (PCOS), an intricate and multifaceted metabolic-endocrine disruption that typically influences 6–20% of women of reproductive age and accounts for 70–80 percent of all occurrences of infertility, is globally among the leading factors responsible for infertility [1]. The hallmarks of PCOS include multisystem endocrinopathy and associated metabolic abnormalities. PCOS is defined by hyperandrogenism (HA), along with cystic ovaries. Even though the precise origin of PCOS is unknown, research points to a connection between the condition and metabolic problems such as obesity, insulin resistance (IR), and type-2 diabetes (T2DM). Because of genetic susceptibility, societal factors, dietary habits, and sedentary lifestyles, the pathobiology of this condition is more pronounced in adults and children. This causes obesity, which negatively affects factors related to the fertility of women, particularly in young women [2]. As the precise etiology of PCOS is unknown and the diagnostic criteria are currently insufficient, there are numerous undiagnosed and untreated PCOS cases in women. Statistics show that seventy-five percent of PCOS-afflicted women see their doctors without a diagnosis [3]. Therefore, its accurate diagnosis and treatment strategy can be achieved by a deeper understanding of its pathological and physiological mechanisms. Cardiac events, type-2 diabetes, obesity, and IR continue to have an impact on its etiology, along with other endocrine and metabolic diseases [4] (Zafar et al., 2019). Numerous types of research have shown that metabolic problems are 3–4 times more common in females with PCOS compared to healthy populations. Researchers have observed that IR activates the ovaries, causing hyperinsulinemia (HI) and resulting in decreased aromatase activity (which poses an issue in balancing sex steroids), an increase in circulating androgens, increased obesity, and worsened IR [5]. These factors all contribute to anovulation, leading to infertility, cardiovascular problems, and T2DM [3]. 

Obesity affects infertility beyond PCOS, exacerbates its metabolic and reproductive abnormalities, and therefore is linked to anywhere between 44 and 61 percent of PCOS cases [6]. The potential of the adipose tissue to generate adipocytokines, including adiponectin, which has glucometabolic, as well as lipid-regulating, anti-inflammatory, and antioxidant activities, makes it an endocrine organ that controls metabolic processes [7]. The decrease in adiponectin release in PCOS demonstrates that adipose tissue hyperplasia and malfunction are involved with the origin of PCOS [8]. Additionally, it has been demonstrated that higher paternal adiponectin transcription provides defense over adipose tissue malfunction [9]. The receptors modulating adiponectin’s activities are widely distributed throughout the hypothalamic–pituitary–ovarian axis and reproductive system, permitting the hormone’s steroidogenic modulatory effects, as well as its metabolic actions in these tissues [10,11]. 

The concept was suggested by recent research [12] that such an adipocytokine might well be involved in the metabolic abnormalities caused by obesity or problems associated with obesity, including polycystic ovarian syndrome. Unfortunately, it is still unclear exactly how these adipocytokines function. Determining whether adiponectin interacts with the endocrine and metabolic issues that accompany PCOS may be helpful in identifying or treating such a complex, reproductive illness. Among the most popular therapies is one that involves the use of an hCG injection in conjunction with clomiphene citrate [13]. Clomiphene users with PCOS experience better reproductive function and menstruation and improved glucose metabolism [14]. Clomiphene can have adverse effects on endometrial thickness because of its chemical resemblance to estrogenic substances [14]. As the long-term usage of conventional medicines has a range of negative effects, experts now advise patients to switch to medicinal herbs, as they have fewer or no side effects.

Chlorogenic acid (CGA), a polyphenolic molecule that is frequently present in a variety of plants, is particularly present in green coffee beans, which have a weight-based CGA concentration of between 5 and 12 percent [15]. People frequently ingest it, and it can be found in many foods and beverages. It is primarily present in vegetables such as potatoes and fruits, including apples, apricots, plums, tomatoes, and cherries. The most popular beverages with high CGA contents are wine, coffee, and tea [16]. Numerous researchers have sought to look into the nutritional advantages and physiological consequences of CGA because it is present in many meals and liquid beverages. Numerous studies have demonstrated that CGA can lessen oxidative stress. They have all demonstrated that it can be used to produce outcomes such as anticancer activity, cardio protection, and maybe even neuroprotection [17]. Evidence suggests that chlorogenic acid contains a variety of effects, including neuroprotective, neurotrophic, and antioxidant properties [18]. CGA’s anti-inflammatory properties have been investigated in a variety of disease models. The NF-B pathway, which activates genes to produce pro-inflammatory cytokines and adhesion molecules, was downregulated in raw macrophages in a mouse model of retinal inflammation, greatly reducing the inflammation caused by endotoxins [19]. Studies have recently concentrated on the role of chlorogenic acid in lipid and glucose metabolism, apart from its significant antioxidative properties. According to reports, CGA inhibited glucose-6-phosphate translocase, which decreased the glucose amount that was transported through the intestines [20]. CGA, however, boosts glucose tolerance and increases glucose absorption, suggesting possible antidiabetic action. Additionally, in rats fed with a high-cholesterol diet, it was observed that CGA reduced plasma and liver lipid levels [21]. Additionally, the metabolic effects of polycystic ovarian syndrome are the cause of all these situations [22]. Hence, the effect of CGA on polycystic ovaries is examined in our study. Our study’s major goal is to examine how chlorogenic acid affects mice with endocrine and metabolic problems brought on by letrozole-induced PCOS. Additionally, we investigate how chlorogenic acid affects the amounts of androgens and adiponectin in the blood. The study further investigates the impact of adiponectin in PCOS-related metabolic and endocrine diseases. Lastly the protein–protein interaction and gene ontology of adiponectin are also analyzed using bioinformatics approach. 

## 2. Methods and Materials

### 2.1. Chemicals

Letrozole and chlorogenic acid (CGA) were purchased from Sigma Aldrich and Sun Pharma, respectively. ELK biotechnology ELISA kits were used in this study and were bought from ELK biotechnology, Wuhan, China, for analysis of hormones. Analytical-grade chemicals were used in addition during the investigation.

### 2.2. Animals

The method was as follows: Eighteen mature Parkes strain mice (4–5 weeks old) weighing 18–21 g were at random separated into three groups with six mice each. Group 1 served as the control and was provided with water and a typical chow food for 81 days; Group II was given letrozole (LETZ) dissolved in normal saline water (0.9%) orally using an oral gavage at a dose of 6 mg/kg bw for 21 days to induce PCOS, followed by 60 days without treatment; Group III was given LETZ (6 mg/kg) for 21 days, followed by oral gavage treatment with CGA (50 mg/kg bw orally daily) for 60 days. Serum was subsequently used for biochemical and hormonal examination. Blood samples from all mice were acquired via retro-orbital venous sinus puncture 24 hours after 81 days of trial. Cervical dislocation resulted in the death of every mouse. Ovaries from all mice were taken out, and adipose fat was cleansed for further biochemical histological studies. All experimental protocols were approved by the institutional ethics committee at Barkatullah University in Bhopal.

### 2.3. Biochemical Studies

The examination of serum hormones was conducted as follows: On day 82 of the trial, to obtain blood, a retro-orbital venous sinus puncture was performed. Following centrifugation, serum was separated and stored until use. The employed ELISA kits were built on competitive inhibition enzyme immunoassay methodology. A specific protein was precoated on a microplate well (MW) included in the kits. Biotin-conjugated antibodies specific for LH, testosterone, estrogen, FSH, and progesterone were then added to the appropriate MW after adding standards or samples. Avidin-horseradish peroxidase (HRP) was then added to each MW, followed by an addition of TMB substrate solution and incubation. A 450 ± 10 nm ELISA reader was utilized to determine the color shift once the enzyme–substrate reaction was stopped using stop solution available in the kits.

Serum insulin and fasting blood glucose were determined as follows: With the aid of readily available commercial kits, serum fasting blood sugar (FBG) levels were measured calorimetrically (Elab science), while serum insulin was measured using an ELISA kit purchased from ELK biotechnology, China.

Serum lipid profiles were determined as follows: With the aid of readily available commercial kits, triglycerides (TGs), high-density lipoprotein (HDL), and total cholesterol (TC) were calorimetrically measured. Very-low-density lipoprotein (VLDL) and low-density lipoprotein (LDL) were measured indirectly using Friedewald’s equation. 

#### Antioxidant Assay 

The lipid peroxidation assay was conducted as follows: The quantity of malonaldehyde (MDA) was measured to determine how much lipid peroxidation (LPO) occurred in the ovaries [23]. We dissected the ovaries in ice-cold normal saline using a glass homogenizer, and ten percent homogenate tissue was prepared. Following homogenization of the ovarian tissue, ten minutes was spent centrifuging the sample at 3000× *g* rpm. Two hours of incubation at 37 °C was spent in a milliliter of supernatant. Then, five minutes was spent centrifuging the sample at 2000× *g* rpm, following proper mixing of samples with one milliliter of 10% tris hydrochloric acid (TCA). An amount of 1 mL of the supernatant was thoroughly combined before being put in a boiling water bath for ten minutes; an equal volume of 0.67 percent 2-thiobarbituric acid TBA was added. After being refrigerated, in order to dilute the samples, 1 mL of distilled water was used. Optical density measurements were made at 535 nm with a spectrophotometer. The information was estimated as nanomoles per gram of tissue.

The reduced glutathione assay was conducted as follows: The concentrations of tissue glutathione (GSH) were measured using a technique known as 5, 50-dithiobis-2-nitrobenzoic acid (DTNB) [24], which included adding and gently mixing 1 cc of 50% TCA after homogenizing the tissue in EDTA 0.02 (5–8 mL), diluting in two milliliters of ice-cold distilled water, and fifteen minutes of spinning at 6000 rpm. To create 1 mL of supernatant, 100 mL of DTNB and 2 mL of tris buffer (0.4 M, pH 8.9) were mixed (0.1 M). Optical density was observed using a spectrophotometer at 410 nm. We calculated the data as nmol/g.

Superoxide dismutase was measured as follows: To measure the activity of superoxide dismutase (SOD), we used the Marklund and Marklund technique [25]. Amounts of 100 mL of pyrogallol and 2.9 mL of tissue homogenate supernatant (10%) were used to evaluate absorbance at 420 nm for three minutes (0.2 mM). We calculated SOD in units per gram of tissue.

### 2.4. Plasma Adiponectin

Adiponectin in plasma was tested using an ELISA kit based on the sandwich ELISA approach acquired from Elabscience Biotechnology Inc. (Wuhan, Hubei, China).

### 2.5. Inflammatory Cytokines

Inflammatory cytokines in plasma were tested using an ELISA kit based on the sandwich ELISA approach acquired from Elabscience Biotechnology Inc.

### 2.6. Reverse Transcription and Real-Time PCR

With the use of TRI Reagent (Invitrogen), total RNA was extracted as directed by the supplier, and a cDNA Synthesis Kit was used to create cDNA from 1 µg total RNA (Invitrogen). To carry out RT-PCR, SYBR green and real-time PCR were employed. The mRNA values were calculated by fitting a standard curve to each gene’s related expression levels. The Table 1 addendum data contain a list of the primers utilized for this study. As an internal control , actin was used (Table 1).

### 2.7. Histological Assessment of Ovaries

Hematoxylin and eosin (H&E) stains were applied after the ovaries were sectioned at a thickness of 5 µm and fixed in 10% Bouin’s fixative for 24 h.

### 2.8. Statistical Analysis

To ascertain the significance of the parameters, a one-way analysis of variance (ANOVA) was employed, followed by a post hoc analysis using Tukey’s multiple comparison test using Graph Pad Prism 8. The mean standard deviations of the data are displayed. To define statistically significant, more significant, and extremely significant differences, *p* values of 0.05, 0.01, and 0.001 were employed, respectively.

### 2.9. Bioinformatics Analysis of Adiponectin

#### 2.9.1. Protein–Protein Interaction of Adiponectin 

The Search Tool for the Retrieval of Interacting Genes (STRING database ver.11.0b), a biological database designed to develop and investigate functional interactions among proteins, was used to establish an adiponectin protein–protein interaction (PPI) network with a confidence level of 0.7. The PPI network was further investigated and visualized using Cytoscape (version 3.8.2) [26]. The Molecular Complex Detection (MCODE) plug-in for the Cytoscape software was included to help identify the PPI network’s notable modules. The PPI network’s top 5 HUB nodes were extracted using the cytohubba plugin [27]. 

#### 2.9.2. Gene Ontology and Pathway Analysis

Gene ontology (GO) of the adiponectin gene (*Adipoq*) was carried out using the Enrichr web platform [28]. The gene ontology terms, viz., molecular functions (MFs), biological processes (BPs), and cellular compartments (CCs), of adiponectin were analyzed, and results with *p* < 0.05 were statistically considered significant. Also, the KEGG analysis was done using ENRICHR database. 

## 3. Results

### 3.1. Effect of Chlorogenic Acid (CGA) on Body Weight, Fasting Blood Glucose, Insulin Concentration, and Changes in Estrous Cyclicity in LETZ-Induced PCOS Mice

**Figure 1:**
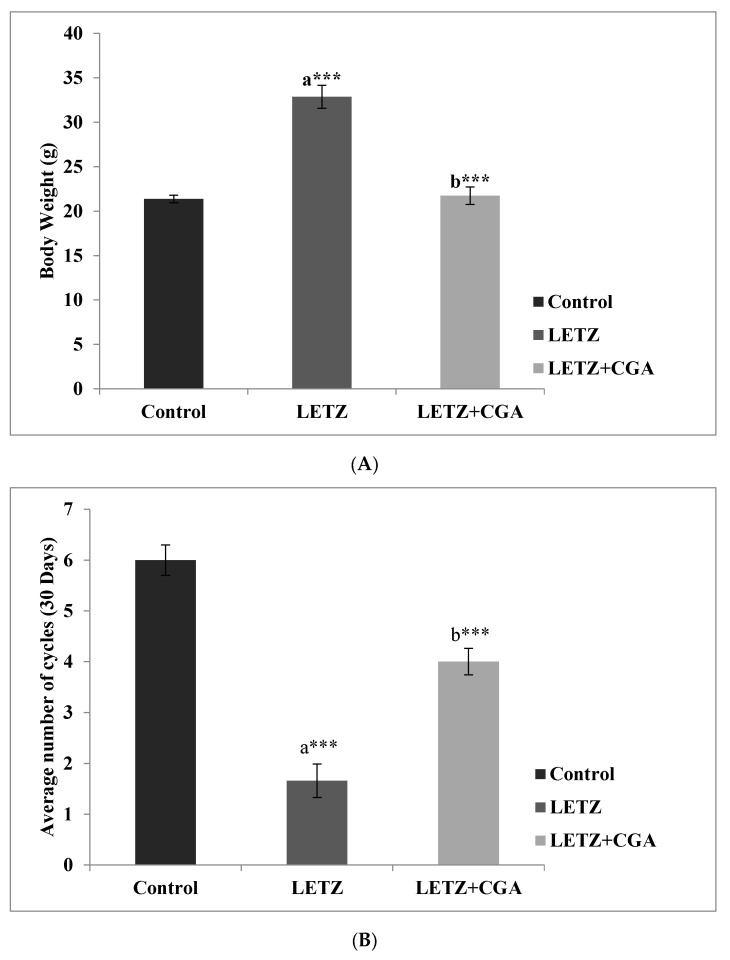

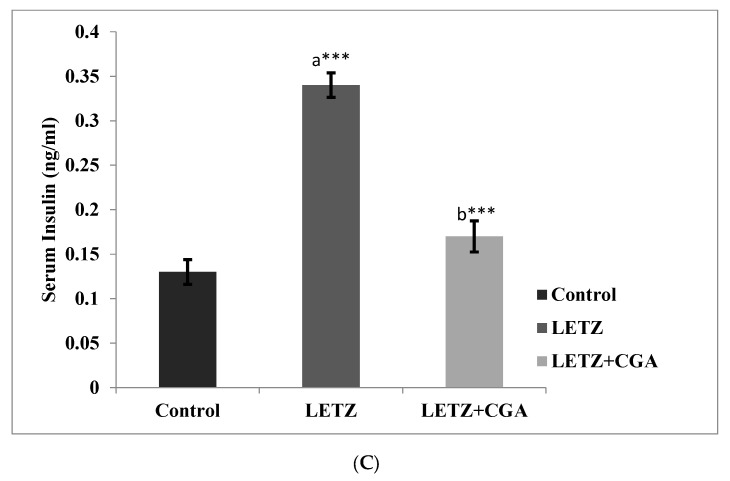
(**A**) Impact of CGA on ovary body mass. PCOS: letrozole (6 mg/kg); control: normal saline; PCOS with CGA: LETZ at 6 mg/kg and CGA at 50 mg/kg; a = PCOS versus control; b = LETZ versus LETZ with CGA. With *n* = 6 per group, all values are expressed as means with standard errors. *** *p* < 0.001; g = grams; CGA = chlorogenic acid; LETZ = letrozole. (**B**) Effect of CGA on estrous cycle in mice with PCOS. Control: normal saline; PCOS: letrozole (6 mg/kg); PCOS with CGA: LETZ at 6 mg/kg and CGA at 50 mg/kg; a = PCOS versus control; b = LETZ versus LETZ with CGA. With *n* = 6 per group, all values are expressed as means with standard errors. *** *p* < 0.001; CGA = chlorogenic acid. **(C)** Effect of CGA on serum insulin levels in mice with PCOS. Control: normal saline; PCOS: letrozole (6 mg/kg); PCOS with CGA: LETZ at 6 mg/kg and CGA at 50 mg/kg; a = LETZ versus control; b = LETZ versus LETZ with CGA. With *n* = 6 per group, all values are expressed as means with standard errors. *** *p* < 0.001; CGA = chlorogenic acid.

We found that LETZ treatment resulted in a significant increase (*p* < 0.001) in body mass. However, LETZ-treated mice treated with both LETZ and CGA indicated a substantial decrease in their weight (*p* < 0001). The results are shown in Figure 1A. In contrast to the typical control mice, the LETZ-treated mice displayed irregular estrous cyclicity, and we discovered a continuous diestrus state that led to longer estrous cycles. However, following CGA therapy, the estrous cycle was regularized, returning the duration to normal (Figure 1B). We also observed that LETZ treatment resulted in a significant increase (*p* < 0.001) in FBG and insulin levels. However, PCOS mice treated with LETZ and CGA for 60 days showed a significant decrease (*p* < 0.001) in FBG and insulin concentration. The data are displayed in Figure 1C and Table 2.

### 3.2. Effect of CGA on Hormone Levels in Mice with PCOS

**Figure 2:**
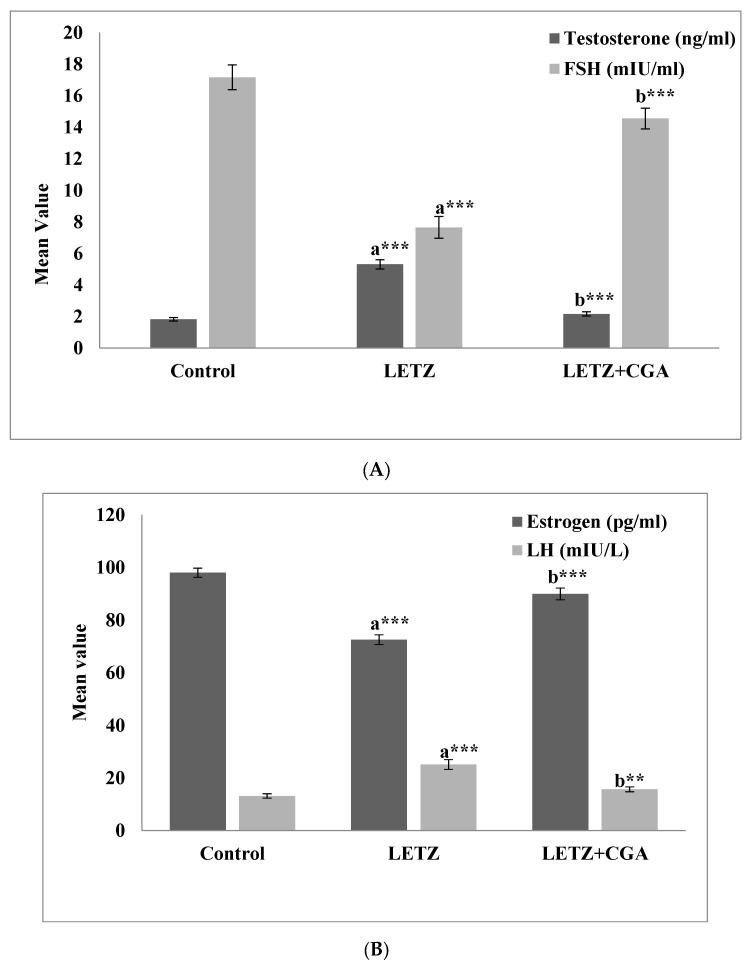

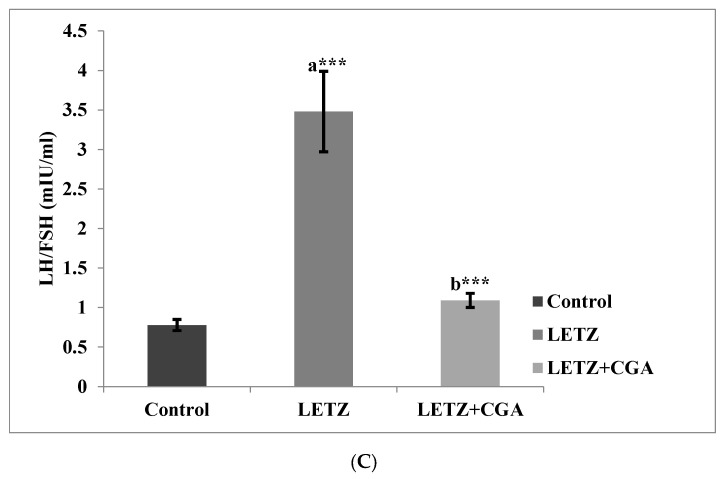
(**A**) Effect of CGA on serum testosterone and FSH levels in mice with PCOS. Control: normal saline; PCOS: letrozole (6 mg/kg); PCOS with CGA: (LETZ at 6 mg/kg and CGA at 50 mg/kg); a = PCOS versus control; b= PCOS versus PCOS with CGA. With *n* = 6 per group, all values are expressed as means with standard errors. *** *p* < 0.001; CGA = chlorogenic acid. (**B**) Effect of CGA on serum estrogen and LH levels in mice with PCOS. Control: normal saline; PCOS: letrozole (6 mg/kg); PCOS with CGA: LETZ at 6 mg/kg and CGA at 50 mg/kg; a = LETZ versus control; b = LETZ versus LETZ with CGA. With *n* = 6 per group, all values are expressed as means with standard errors. *** *p* < 0.001; ** *p* < 0.01; CGA = chlorogenic acid. (**C**). Effect of CGA on serum LH and FSH ratio levels in mice with PCOS. Control: normal saline; PCOS: letrozole (6 mg/kg); PCOS with CGA: LETZ at 6 mg/kg and CGA at 50 mg/kg; a = LETZ versus control; b = LETZ versus LETZ with CGA. With *n* = 6 per group, all values are expressed as means with standard errors. *** *p* < 0.001; ** *p* < 0.01; CGA = chlorogenic acid; LETZ = letrozole.

The results revealed that LETZ treatment resulted in a significant increase (*p* < 0.001) in testosterone and LH levels and a decrease (*p* < 0.001) in the levels of estrogen and FSH. However, mice with LETZ-induced PCOS treated with LETZ and CGA for 60 days normalized these changes. Moreover, the LH/FSH ratio was found higher in LETZ-treated mice, which decreased after treatment with LETZ and CGA. The results are shown in Figure 2A–C.

### 3.3. Impact of CGA Treatment on Lipid Profiles in PCOS Mice

The results revealed that LETZ treatment resulted in a significant increase (*p* < 0.001) in cholesterol, TG, LDL, and VLDL levels while decreasing HDL levels. However, after treatment with CGA, these changes were reversed significantly, and the results are shown in Table 2.

### 3.4. Impact of CGA on Lipid Peroxidation of Antioxidants 

**Figure 3:**
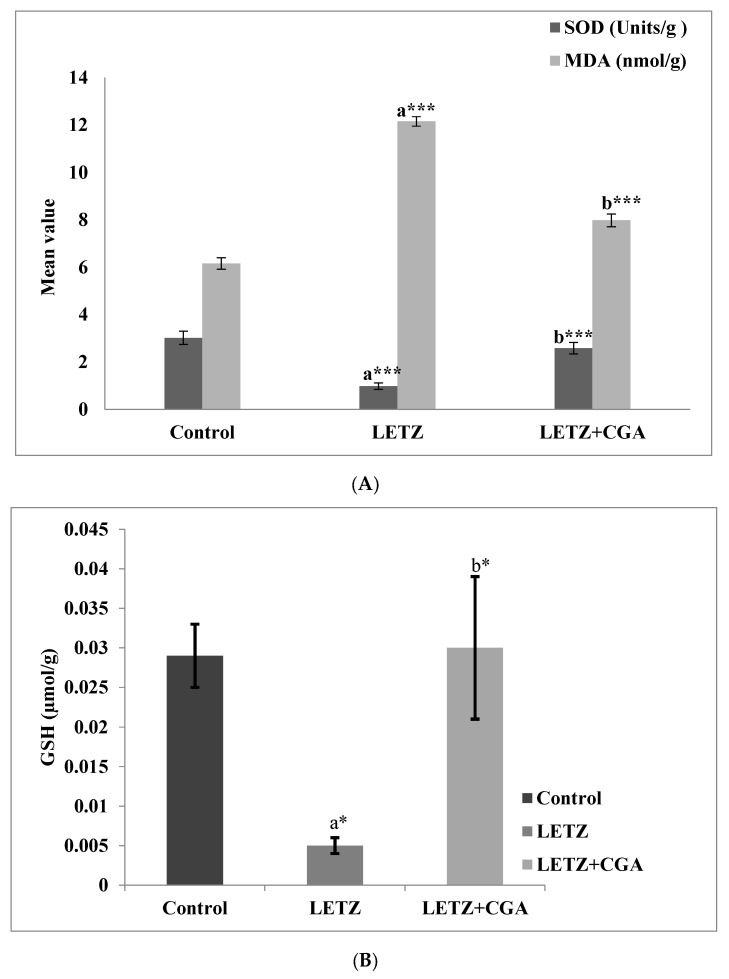
(**A**) Effect of CGA on MDA and SOD levels in mice with PCOS. Control: normal saline; PCOS: letrozole (6 mg/kg); PCOS with CGA: LETZ at 6 mg/kg and CGA at 50 mg/kg; a = LETZ versus control; b = LETZ versus LETZ with CGA. All values displayed are expressed as means ± SEM with 6 animals in each group. *** *p* < 0.001; CGA = chlorogenic acid; LETZ = letrozole. (**B**) Effect of CGA on GSH levels in mice with PCOS. Control: normal saline; PCOS: letrozole (6 mg/kg); PCOS with CGA: LETZ at 6 mg/kg and CGA at 50 mg/kg; a = LETZ versus control; b = LETZ versus LETZ with CGA. With *n* = 6 per group, all values are expressed as means with standard errors. * *p* < 0.05; CGA = chlorogenic acid; LETZ = letrozole.

The mice that were treated with letrozole showed a significant decrease (*p* < 0.001) in antioxidant capacity compared to the control group. Treatment of mice with PCOS using CGA significantly increased their antioxidant capacity, and the results are shown in Figure 3A,B.

### 3.5. CGA Administration Treatment Reduces Inflammatory Cytokines in PCOS Mice and Effects the Expressions of Genes Related to Hormones

**Figure 4:**
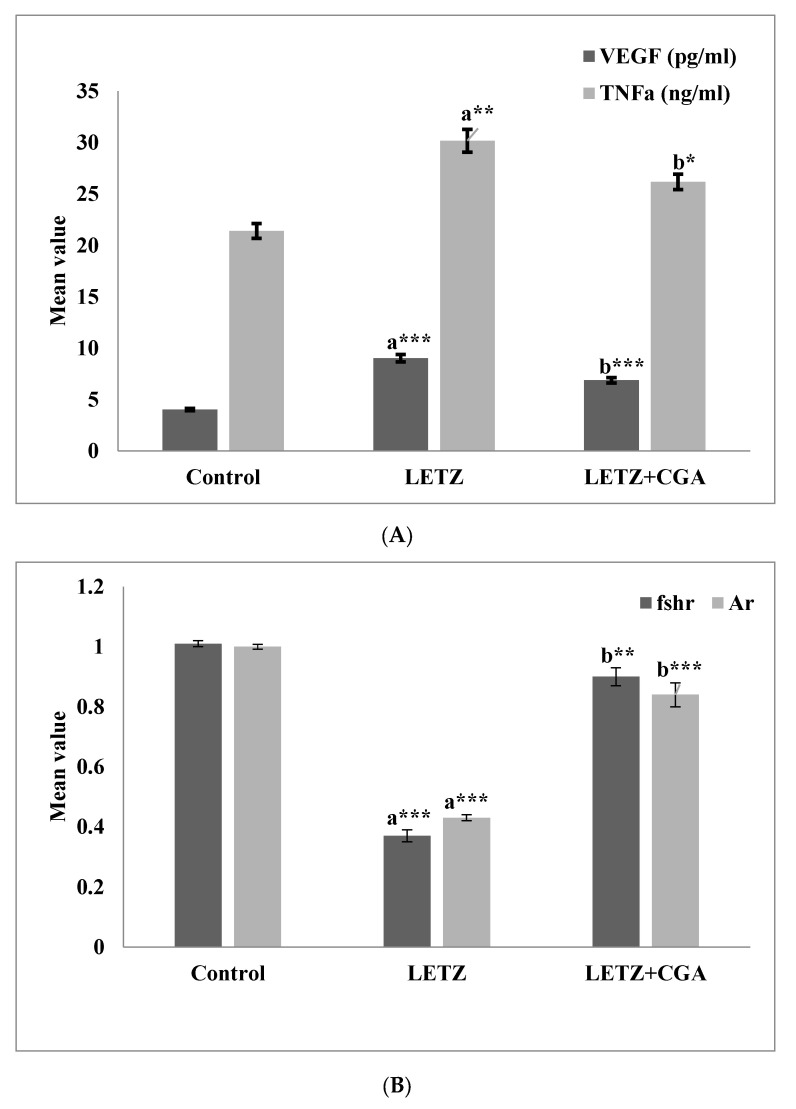

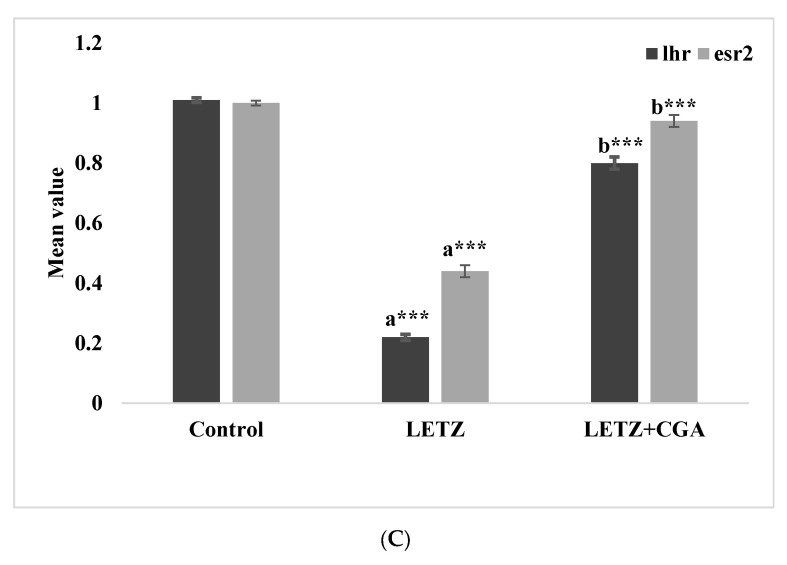
(**A**) Effect of CGA on VEGF and TNFɑ levels in mice with PCOS. Control: normal saline; PCOS: letrozole (6 mg/kg); PCOS with CGA: LETZ at 6 mg/kg and CGA at 50 mg/kg; a = LETZ versus control; b = LETZ versus LETZ with CGA. With *n* = 6 per group, all values are expressed as means with standard errors. *** *p* < 0.001, ** *p* < 0.01, and * *p* < 0.05; CGA = chlorogenic acid; LETZ = letrozole. (**B**) Effect of CGA on fshr and Ar expressions in mice with PCOS. Control: normal saline; PCOS: letrozole (6 mg/kg); PCOS with CGA: LETZ at 6 mg/kg and CGA at 50 mg/kg; a = LETZ versus control; b = LETZ versus LETZ with CGA. With *n* = 6 per group, all values are expressed as means with standard errors. *** *p* < 0.001; ** *p* < 0.01; CGA = chlorogenic acid; LETZ = letrozole. (**C**) Effect of CGA on lhr and esr2 expressions in mice with PCOS. Control: normal saline; PCOS: letrozole (6 mg/kg); PCOS with CGA: LETZ at 6 mg/kg and CGA at 50 mg/kg); a = LETZ versus control; b = LETZ versus LETZ with CGA. With *n* = 6 per group, all values are expressed as means with standard errors. *** *p* < 0.001; CGA = chlorogenic acid; LETZ = letrozole.

In our study, we assessed the role of CGA on inflammatory cytokines, such as TNF-ɑ and VEGF, both of which were increased (*p* < 0.001) in the LETZ-induced PCOS mice compared to the control mice. Treatment of PCOS mice with CGA indicated a substantial decrease in their levels, and the results are shown in Figure 4A. By using specific primers, we performed RT-PCR for the receptors of androgen, FSH, LH, and estrogen, and we observed that Ar, esr, and lhr were downregulated in LETZ-induced PCOS mice, while fshr was upregulated. Treatment of LETZ-induced PCOS mice with CGA normalized these changes, as shown in Figure 4B,C.

### 3.6. CGA Treatment Increases the Levels of Adiponectin and Adipo-R1 Expression in PCOS Mice

**Figure 5:**
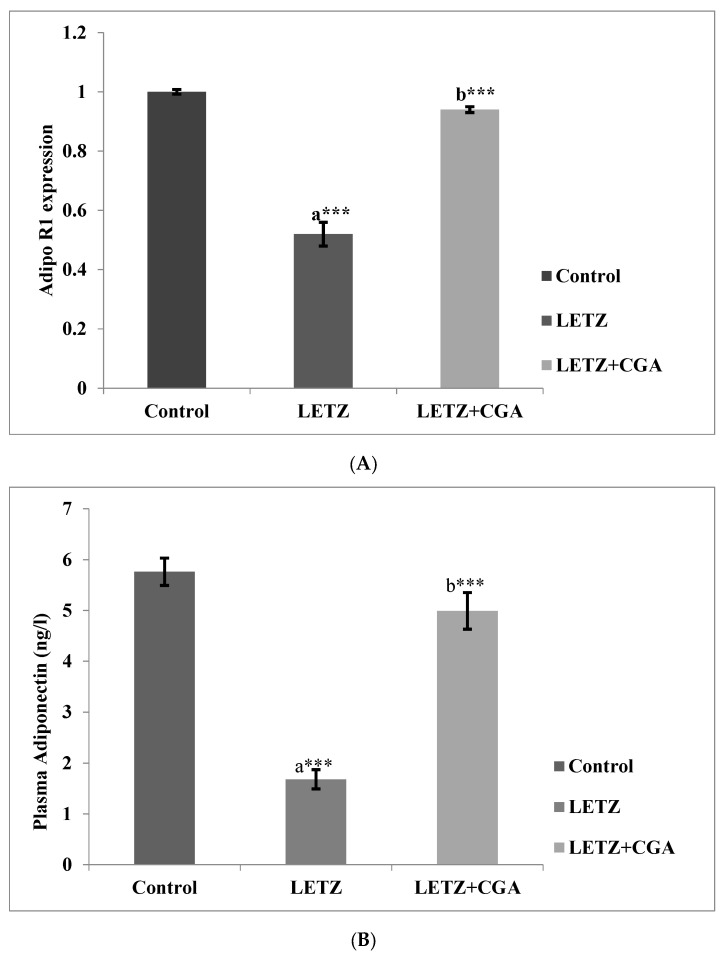
(**A**) Effect of CGA on Adipo r1 expression in mice with PCOS. Control: normal saline; PCOS: letrozole (6 mg/kg); PCOS with CGA: LETZ at 6 mg/kg and CGA at 50 mg/kg; a = LETZ versus control; b = LETZ versus LETZ with CGA. With *n* = 6 per group, all values are expressed as means with standard errors. *** *p* < 0.001; CGA = chlorogenic acid; LETZ = letrozole. (**B**) Effect of CGA on adiponectin levels in mice with PCOS. Control: normal saline; PCOS: letrozole (6 mg/kg); PCOS with CGA: LETZ at 6 mg/kg and CGA at 50 mg/kg); a = LETZ versus control; b = LETZ versus LETZ with CGA. With *n* = 6 per group, all values are expressed as means with standard errors. *** *p* < 0.001; CGA = chlorogenic acid; LETZ = letrozole.

In our study, we observed the plasma levels of adiponectin and adipo r1 expression in the ovarian tissue, both of which were decreased significantly (*p* < 0.001) in LETZ-treated mice. Treatment of mice with PCOS using CGA caused a significant increase (*p* < 0.001) in their levels, and the results are shown in Figure 5A,B.

### 3.7. Protein–Protein Interactions of Adiponectin

**Figure 6:**
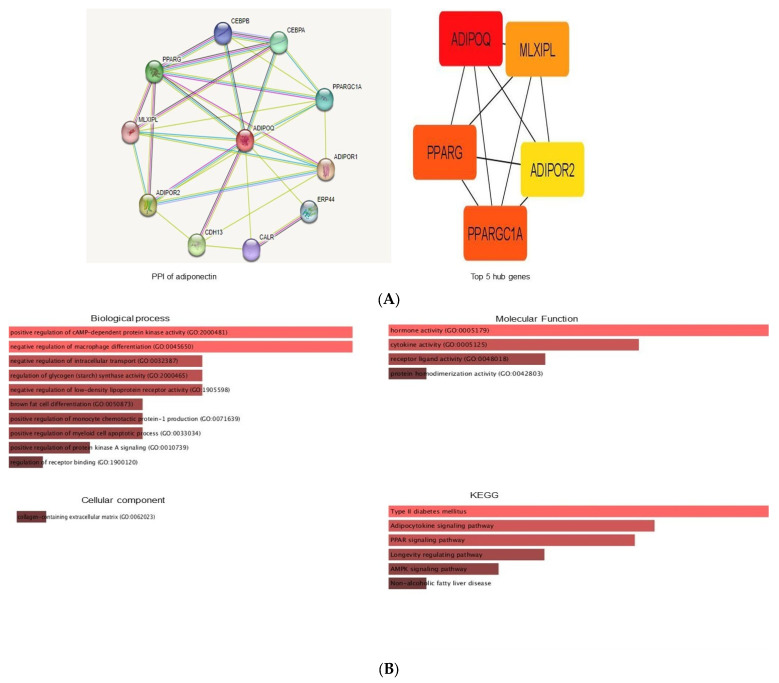
(**A**) PPI analysis of Adipoq. PPI constricted module of Adipoq using STRING and top 5 hub genes of PPI network identified using cytohubba. (**B**) Gene ontology functional analysis of the BP, MF, and CCanalyses of adiponectin and KEGG Pathway analysis.

Co-expressions of 11 genes (nodes) along 30 protein–protein links (edges) were connected to establish a web of protein–protein interactions. Additionally, the generated PPI network showed a median node degree of 5.45, an expected number of edges of 12, an average local clumping correlation of 0.788, and a PPI enriched *p*-value of 1.4 × 10^−5^ (Figure 6A). Cytohubba was employed to determine the five top core genes in the network based on degree scoring, as shown in Figure 6A. Adiponectin (Adipoq), MLX interacting protein like (MLXIPL), peroxisome proliferator-activated receptor gamma Coactivator 1- alpha (PPARGC1)A peroxisome proliferator-activated receptor gamma (Pparg), and adiponectin receptor 1 (Adipor1) were the leading networking genes.

### 3.8. Gene Ontology and KEGG Pathway Analysis

The GO enrichment study was carried out using Enrichr. Compared to MFs and CCs, the gene ontology study revealed that BPs were significantly enriched 8. Amongst BPs, adiponectin was discovered to be implicated in the negative regulation of glycogen synthase activity and low-density lipoprotein receptor activity, as well as in the positive regulation of the myeloid cell apoptotic process and protein kinase A (Figure 6B). The KEGG pathway study showed that Adipoq is associated with type II diabetes mellitus, adipocytokine signalling pathway, PPAR signalling pathway etc (Figure 6B).

### 3.9. CGA Improves Ovarian Morphology in Mice with Letrozole-Induced PCOS

**Figure 7:**
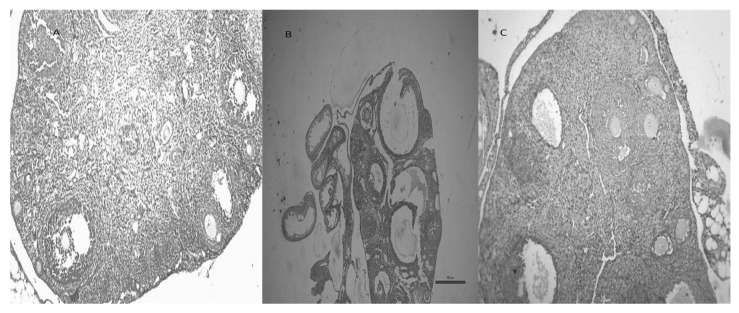
(**A**–**C**) Histopathological photomicrographs of each experimental group’s ovaries (H&E, magnification ×10): (**A**) control, (**B**) LETZ, and (**C**) LETZ with CGA.

The histopathological analysis of an ovary portion exhibited the shapes of the granulosa cells, secondary follicles, and oocytes. In contrast to the control group of mice, the LETZ-induced PCOS mice displayed ovarian follicle degeneration, follicular cyst formation, and distorted granulosa cells, along with an absence of oocytes. On the other hand, the ovarian tissue of the LETZ-and-CGA-treated group showed normal granulosa cells and an antral cavity with a clearly isolated oocyte (Figure 7).

## 4. Discussion

PCOS is a diverse and multigenic disorder that is really very expensive for both the patient, as well as for society. In spite of certain other challenges, including heart problems and insulin resistance, clinical manifestations of PCOS often include hirsutism, abdominal obesity, and acne, which are linked to high androgens and cause women to experience stress and anxiety. Pharmacological innovations having fewer side effects deserve special consideration. By controlling the flows of testosterone and adiponectin, we demonstrated that chlorogenic acid (CGA) restored both metabolic and endocrine problems associated with LETZ-induced PCOS. We were capable of proving that letrozole contributed to PCOS, which is categorized by overweight and polycystic ovaries, by employing female laboratory mice of the Parke’s strain. In addition to these, there were also enhanced insulin and hyperlipidemia. LETZ-induced PCOS mice had lower concentrations of adiponectin, increased concentrations of inflammatory cytokines, and diminished ovarian cellular antioxidant capabilities. After CGA therapy, these changes were alleviated.

Leukocytes dominated within the letrozole group, suggesting a continuous diestrus stage of vaginal smears [29], validating the establishment of PCOS in an ongoing study. Interestingly, letrozole blocks the conversion of androgens to estrogens, which results in HA. Estrous cycle disruption, an increase in reproductive organ and body weight, and testosterone upregulation are all effects of enhanced androgens [30]. We noticed that LETZ-treated mice showed abnormalities in the estrous cycle, as well as increased body and ovarian mass, and these findings are consistent with past studies. After CGA treatment, the estrous cycle reverted to normal, and both body weight and ovary weight decreased. 

In addition to type-2 diabetes, women experiencing PCOS also develop metabolic and other related issues, such as IR, poor glucose tolerance, and adiposity [31]. Our findings revealed that LETZ-induced PCOS in mice caused elevation in FBG and insulin levels, suggesting IR and hyperglycemia (HG), two vital features of metabolic abnormalities [32]. Additionally, oxidative stress (OS), especially in metabolic syndrome, can lead to organ failure, such as reproductive failure, either functionally or structurally. HI and HG are key metabolic events that trigger an inflammatory cascade. In a manner comparable to this, the enhanced insulin observed in mice treated with LETZ might even influence the functions of metabolically active tissues influenced by insulin, including ovaries, resulting in metabolic abnormalities in the reproductive system.

**Figure 8:**
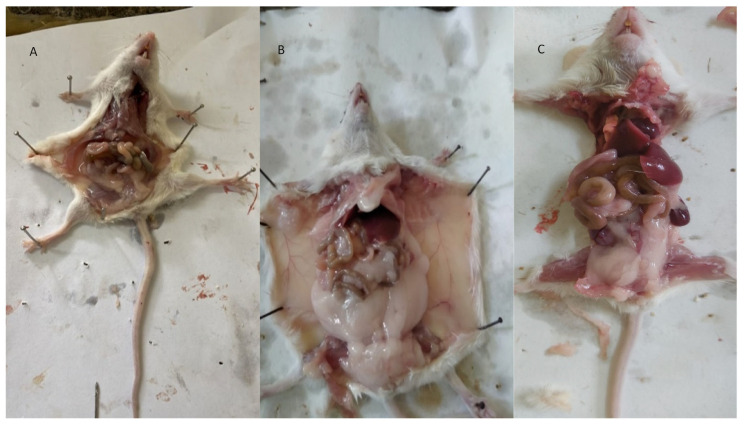
Comparison of visceral fat reduction in the CGA-treated group to that in the PCOS-induced LETZ group. The CGA-treated group had less fat tissue in the abdominal cavity, especially around the uterus and ovaries. The image in (**A**) shows visceral fat displayed in control group, while (**B**) represents LETZ and (**C**) represents LETZ with CGA.

Moreover, earlier research proved that IR increased adiposity, a crucial contributor to obesity in metabolic and other associated disorders [33]. Nearly 50% of PCOS individuals have abdominal fat (visceral fat) build-up. Thirty to sixty percent of people have obesity at some point in their lives, making it a common sign of PCOS [34]. In patients with PCOS, insulin resistance is likely caused by abdominal obesity [35]. In this study, PCOS mice showed an increase in visceral fat similar to earlier studies, and we observed a decrease in visceral fat in the PCOS mice that were treated with CGA (Figure 8). A previous study reported a decrease in visceral fat in rats that were given a diet heavy in fat and medicated with CGA [36].

Patients with PCOS who have hyperinsulinemia experience increased catecholamine-induced lipolysis in adipocytes, which raises blood free fatty acids and, subsequently, induces dyslipidemia. The liver creates more free fatty acids as a result, which increases the levels of VLDL and TGs in the blood. In this study, we discovered that letrozole-treated mice had higher TC, TG, HDL, and VLDL levels and lower LDL levels than the control group. Conversely, treatment with CGA revealed a marked drop in these concentrations, as well as a rise in LDL cholesterol levels. Similar effects of CGA were discovered in earlier research [36].

Evidence suggests that tissues of the ovary are prompted to synthesize androgens by inhibiting the action of aromatase [5,37]. Anastrozole, an aromatase inhibitor, was associated with suppressing estrogen, which causes insulin resistance and decreased peripheral glucose clearance when given to healthy people or when treating cancer [38]. To confirm the impact of CGA on changes in hormones, we examined the serum levels of hormones. Serum androgen levels were elevated in PCOS-affected mice, which was the most consistent hormonal trait. Increased testosterone and LH and reduced estrogen, FSH, and progesterone have also been noted in PCOS-afflicted mice [39,40]. In this investigation, in contrast to levels in PCOS-afflicted mice, CGA decreased serum testosterone, LH, and LH/FSH concentrations. LH levels above normal and elevated LH and FSH ratios may be used as indicators of PCOS among females [40]. In addition, CGA treatment in mice with PCOS significantly reduced serum testosterone and LH levels. PCOS illness may benefit from the decreasing of these elevated testosterone levels since it has already been shown that a high androgen level contributes to the etiology of PCOS [41,42]. Contrary to testosterone, LETZ-treated mice had lower serum estrogen, progesterone, and FSH levels, and the decline was associated with mid- or early follicular growth, as well as the creation of the morphology of follicles in the ovary [43]. Here, it was observed that PCOS mice had decreased concentrations of Ar and Esr1 mRNA levels, but in our research, CGA therapy reversed this downregulation in mice with PCOS. Ar and Esr1 transcripts have been shown to serve a function of proliferation in follicular growth [44,45], and elevated Ar levels can facilitate granulosa cell proliferation and differentiation [46]. Fshr moderately controls follicle growth during the baseline follicle growth phase by working in synergy with other stimulating substances, such as androgens [47]. Lhr is also present on the surfaces of granulosa and theca cells, and levels of Lhr have an impact on ovulation, the development of the corpus lutum, and on the synthesis of additional steroid, such as estrogen, androgen, and progesterone [48]. Such findings led us to discover cystic degeneration of the corpus luteum and follicles in PCOS-affected animals. It is interesting to note that CGA helped to slow the degradation of ovarian follicle development by regenerating the corpus luteum and eliminating cystic follicles.

The most successful treatments for atypical symptoms linked to female reproductive illnesses are hormones, but such treatments have a number of adverse effects, including uterine hemorrhage and hyperplasia [49,50]. Such findings imply that CGA might be an effective medication in treating hormonal imbalances brought on by PCOS. Lower steroid hormone levels in the ovary are correlated with higher numbers of developing follicles and their different shapes [51].

A crucial role of cancer development is known to be played by oxidative stress, which is changed in PCOS. There exists a link between dyslipidemia (DL) and OS [52]. The results of this study suggested that increased lipid peroxidation may be caused by dyslipidemia and an increase in ovarian lipid accumulation, which was characterized by increased MDA. It, thus, ultimately led to a decline in superoxide dismutase (SOD) and GSH in LETZ-induced PCOS mice, hence leading to OS, which in turn contributed to the ovarian tissue injury that precedes infertility in females of child-bearing age. This was visible in the histology of the ovary, with deteriorated follicles and disturbed granulosa cells, oocytes, and antrum. In this investigation, we found that an injection of CGA normalized MDA, SOD, and GSH levels. Previous studies also found a role of CGA in preventing oxidative stress from causing harm [53]. The outcomes of the present investigation further demonstrated that PCOS animals had increased TNF-α compared to control animals. Prior research indicates that both HI and HG caused inflammation, apart from oxidative damage [40], which could account for the increased levels of TNF-ɑ found in mice with LETZ-induced PCOS. Vascular endothelial growth factor (VEGF) is extremely important for pathological, physiological, and developmental angiogenesis, according to Wei et al. [54]. Oxidative stress triggers a state that is pro-inflammatory and leads to both hyperandrogenism and insulin resistance in a feedback loop [55]. According to reports, PCOS women secrete more VEGF as a result [56]. The mechanism is explained by the fact that the VEGF promoter region contains androgen receptor (AR) binding sites. The VEGF gene is activated when androgens bind to these sites. Additionally, the blood of PCOS women has less VEGF receptors, which increases the bioavailability of VEGF, as shown by Artini and colleagues [56]. These outcomes are congruent with the higher VEGF levels observed in the letrozole group. Furthermore, compared to the letrozole-only group, TNF and VEGF levels were reduced when LETZ and CGA were administered.

Importantly, animals with PCOS caused by LETZ had considerably lower circulating adiponectin and Adipo R1 than the healthy control group, which supports earlier research suggesting that, in PCOS patients, adiponectin was a measure of IR [57]. Furthermore, we found that circulating adiponectin was inversely linked with ovarian tissue histology, insulin resistance, FBG, oxidant–antioxidant status, lipid management, and inflammatory markers in LETZ-induced PCOS animals. In various studies, it has been discovered that metabolic disorders and their associated syndromes have reduced adiponectin concentrations [58]. Apart from its effects on metabolism, it was also shown that adiponectin is an agent controlling gametogenesis [59]. It also influences the function of the gonads and GnRH synthesis [60] (Yuan et al., 2016). Because there is a correlation between the quantity of estrogen present in the follicular fluid and the ovarian cells of the dominant follicle, in rodents, follicle predominance and egg quality are connected to adiponectin [61]. 

Additionally, it was demonstrated that recombinant adiponectin increased hormone release, primarily estrogen, in women and rodents at doses of 5–10 g/mL [61]. Therefore, our results implied that adiponectin directly influenced PCOS’s metabolic and endocrine features, and its drop could cause female infertility. Therefore, boosting the circulation of adiponectin may be employed as a remedy to metabolic and, perhaps, endocrine conditions in polycystic-ovarian-syndrome-affected individuals. This is the only research to the best of our knowledge that demonstrates the positive effects of CGA on the endocrine-metabolic issues connected to LETZ-induced PCOS in female mice and the role of CGA regarding adiponectin. However, the results of our study may have therapeutic repercussions for PCOS-affected individuals everywhere. In addition, the protein–protein interactions of adiponectin revealed the top five hub genes, and the gene ontology depicted the role of adiponectin in various processes, such as the negative regulation of glycogen synthase activity and low-density lipoprotein receptor activity and the positive regulation of myeloid cell apoptotic process and protein kinase A.

## 5. Conclusions

To establish a mouse model with symptoms similar to those of people with the illness, letrozole was administered orally to the mice. According to the findings of our investigation, adiponectin directly affected endocrine and metabolic components of PCO, and its drop could cause female infertility. Therefore, boosting the circulation of adiponectin could be employed as a remedy to metabolic and, perhaps, endocrine conditions in polycystic-ovarian-syndrome-affected individuals. In our study, we demonstrated the positive effects of CGA on the endocrine-metabolic issues connected to LETZ-induced PCOS in female mice and role of CGA regarding adiponectin. However, the results of our study may have therapeutic repercussions for PCOS-affected individuals everywhere.

## Figures and Tables

**Table 1:** List and sequence of primers used for the analysis of RT-PCR.

S.No	Gene	Forward Primer (5′ –3′)	Reverse Primer (3′ –5′)
**1**	**Ar**	CTGGGAAGGGTCTACCCAC	GGTGCTATGTTAGCGGCCTC
**2**	**Lhr**	ACACTGCCCTCCAAAGAAAA	CCTCAAAGATGGCGGAATAA
**3**	**Fshr**	CTCATCAAGCGACACCAAGA	GGAAAGGATTGGCACAAGAA
**4**	**Esr2**	AGTAGCCGGAAGCTGACACA	CATGCTGAGCAGATGTTCCA
**5**	**AdipoR1**	CGC TTT CTG CGT ATC GTC TG	CCA ACC TGC ACA AGT TCC CTT
**6**	**Actin**	TACGTCGCCCTGGATTTT	ATGAAAGAGGGCTGGAAGAG

**Table 2:** Impact of CGA on fasting glucose and lipid profiles in PCOS mice.

Groups	Fasting Glucose (mg/dL)	Total Cholesterol (TC) (mg/dL)	Triglycerides (mg/dL)	HDL (mg/dL)	LDL (mg/dL)	VLDL (mg/dL)
**Control**	109.96 ± 1.52	118.56 ± 1.56	121.23 ± 0.99	78.63 ± 0.99	15.68 ± 2.21	24.25 ± 0.9
**LETZ**	153.52 ± 2.48 ^a ***^	155.18 ± 2.91 ^a ***^	153.93 ± 1.51 ^a ***^	48.46 ± 1.87 ^a ***^	75.93 ± 4.11 ^a ***^	30.78 ± 0.3 ^a ***^
**LETZ with CGA**	117.86 ± 3.41 ^b ***^	130.78 ± 2.24 ^b ***^	128.86 ± 1.65 ^b ***^	69.16 ± 1.15 ^b ***^	35.84 ± 3.4 ^b ***^	25.77 ± 0.3 ^b ***^

PCOS: letrozole (6 mg/kg); control: normal saline; PCOS with CGA: LETZ at 6 mg/kg and CGA at 50 mg/kg; a = LETZ versus control; b = LETZ versus LETZ with CGA. All values displayed are expressed as means ± SEM with 6 animals each group. *** *p* < 0.001; g = grams; mg = milligrams; CGA = chlorogenic acid; LETZ = letrozole.

## Data Availability

The authors declare that the protocol and procedures employed were ethically reviewed and approved by the Institutional Ethics and Protocols Committee. Furthermore, all the data are available from the authors and will be made available on request.
